# SARS-CoV-2 pandemic non-pharmacologic interventions temporally associated with reduced pediatric infections due to *Mycoplasma pneumoniae* and co-infecting respiratory viruses in Arkansas

**DOI:** 10.1128/spectrum.02908-23

**Published:** 2024-03-15

**Authors:** Bobby L. Boyanton, Rachel A. Frenner, Ashton Ingold, Lilliam Ambroggio, Joshua L. Kennedy

**Affiliations:** 1 Department of Pathology, Arkansas Children's Hospital, Little Rock, Arkansas, USA; 2 Department of Pathology, University of Arkansas for Medical Sciences, Little Rock, Arkansas, USA; 3 Arkansas Children’s Research Institute, Little Rock, Arkansas, USA; 4 Sections of Emergency Medicine and Hospital Medicine, Children's Hospital Colorado, Aurora, Colorado, USA; 5 Department of Pediatrics, University of Colorado, Aurora, Colorado, USA; 6 Departments of Pediatrics and Internal Medicine, University of Arkansas for Medical Sciences, Little Rock, Arkansas, USA; Ann & Robert H. Lurie Children's Hospital of Chicago, Chicago, Illinois, USA

**Keywords:** severe acute respiratory syndrome coronavirus 2, COVID-19, infection control, social distancing, universal masking, NPI

## Abstract

Non-pharmacologic interventions (NPIs), such as universal masking, implemented during the SARS-CoV-2 pandemic have reduced respiratory infections among children. This study evaluated the impact of NPIs on *Mycoplasma pneumoniae* infections in children, analyzing data from two hospitals in Arkansas and examining age-related differences and co-infections with other respiratory viruses. The study was approved by the Institutional Review Board and included patients (≤18 years) with upper respiratory tract symptoms. Data generated from the FilmArray Respiratory Panel were divided into pre-NPI, NPI, and post-NPI periods for analysis. Overall test positivity rate and positivity rate interval changes were evaluated. Statistical differences were determined by Chi-square (χ^2^ independence) analysis. A total of 100,077 tests were performed, with a statistical increase in testing volume during the NPI and post-NPI periods. The number of positive *M. pneumoniae* tests decreased by 77% (77 to 18) during the NPI period, then increased by 50% (18 to 27) during the post-NPI period. Preschool and elementary school age groups had the highest number of positive tests during the study at 59 (48%) and 40 (33%), respectively. Reduced *M. pneumoniae* infections were consistent across age groups. Co-infections with other respiratory viruses, particularly human rhinovirus/enterovirus, were observed at much lower levels. Pediatric *M. pneumoniae* infections in Arkansas were temporally associated with implementation and discontinuation of NPIs. Specific viral co-infections still occurred, albeit at lower levels during the SARS-CoV-2 pandemic. Because of the slower growth of this bacterium, we expect *M. pneumoniae* infections to return to pre-pandemic levels within approximately 2 years.

**IMPORTANCE:**

Non-pharmacologic interventions (NPIs) effectively curtailed the spread of SARS-CoV-2 and, fortuitously, many other aerosol-transmitted respiratory pathogens. This study included the largest data set of symptomatic, pediatric patients from within the United States spanning a period from November 2017 through December 2023, and encompassed individuals residing in both rural and urban settings. We observed a strong correlation between the implementation and cessation of NPIs with the rate of respiratory infections due to *Mycoplasma pneumoniae* and viral co-infections. These infections are returning to baseline levels approximately 2 years following NPI cessation. This observation was not unexpected since the replication time for viruses is exponentially faster than that of bacteria. The resurgence of *M. pneumoniae* and likely other atypical bacterial pathogens is currently in process. Healthcare providers should strongly consider these pathogens in individuals presenting with respiratory tract illnesses.

## INTRODUCTION

Non-pharmacologic interventions (NPIs), such as universal masking, physical distancing, and school closures, were recommended in March 2020 to mitigate the SARS-CoV-2 pandemic (1). These interventions were implemented to reduce the transmission of the virus and prevent the spread of COVID-19. While NPIs primarily focused on preventing COVID-19 cases, additional benefits, including decreased hospitalizations and deaths, have been observed with other respiratory infections, especially in children. Compliance with NPIs resulted in a significant decrease in respiratory infections among children. Multiple studies have now reported reduced rates of respiratory illnesses, such as the common cold, croup, otitis media, pharyngitis, pneumonia, and sinusitis caused by different viruses and bacteria (2–14). The use of masks has been particularly effective in preventing the spread of respiratory droplets and aerosol particles that can carry infectious agents.

*Mycoplasma pneumoniae* is a bacteria that infects the respiratory epithelium and causes mild to severe upper and lower respiratory tract diseases in children and adults, including community-acquired pneumonia (CAP). It is transmitted through person-to-person contact via aerosols produced during coughing and sneezing. Implementing infection prevention measures such as wearing masks and maintaining physical distance can help reduce the transmission of *M. pneumoniae* and other aerosol-spread infectious agents. During the SARS-CoV-2 pandemic, the widespread adoption of NPIs led to a significant decrease in upper and lower respiratory tract infections, including those caused by *M. pneumoniae* (14–21). This reduction in infections can be attributed to the decreased opportunities for close contact and the adherence to hygiene practices like handwashing and respiratory etiquette.

However, the detection and surveillance of *M. pneumon*iae infections have been challenging due to the lack of standardized diagnostic methods. Diagnostic testing for *M. pneumoniae* includes antigen, culture, nucleic acid amplification (NAA), and serology. However, these methods vary in sensitivity and specificity, making it difficult to accurately track the prevalence of these infections. Consequently, surveillance efforts have been limited, and comprehensive data on the impact of NPIs on *M. pneumoniae* infections have been scarce. Furthermore, *M. pneumoniae* infections pose a diagnostic challenge in differentiating symptoms from other respiratory viral pathogens. Age-related differences are also possible, with younger patients either showing no noticeable symptoms or presenting with coryza and wheezing without fever.

To address this knowledge gap, a study was conducted at Arkansas Children's Hospital (ACH) and Arkansas Children's Northwest (ACNW) to evaluate the impact of NPIs on *M. pneumoniae* infection rates in children. The study aimed to provide valuable insights into the association between NPIs and *M. pneumoniae* infections in children by analyzing the data collected from these two medical facilities. A secondary goal was to assess age-related differences and co-infections with other viruses in the children of Arkansas before, during, and following the COVID-19 pandemic.

## MATERIALS AND METHODS

This retrospective cross-sectional study received approval from the Institutional Review Board of the University of Arkansas for Medical Sciences with waiver of consent and HIPAA authorization (No. 274080). The study included patients aged ≤18 years who presented with upper respiratory tract symptoms and were tested using the BioFire FilmArray Respiratory Panel (FARP; bioMerieux, Durham, NC, USA) from 01 November 2017 to 31 December 2023 at ACH (Little Rock, AR) or ACNW (Springdale, AR). The FARP detected various respiratory pathogens, including adenovirus, coronaviruses (229E, HKU1, NL63, and OC43), human metapneumovirus, human rhinovirus/enterovirus, influenza viruses (A, A/H1, A/H3, and B), parainfluenza viruses (1–4), respiratory syncytial virus, *Bordetella pertussis*, *Chlamydia pneumoniae*, and *M. pneumoniae*. During the study period, the U.S. Food and Drug Administration approved newer versions of the FARP for clinical use. Specifically, *Bordetella parapertussis* and SARS-CoV-2 were included in December 2017 and June 2020, respectively. Patient demographic information, FARP test results, and data on co-infections were collected from the electronic health record system (EPIC, Verona, WI).

In Arkansas, NPIs were implemented in late March 2020, including universal masking, physical distancing, restricted access to public activities, and temporary closure of in-person school and daycare facilities. Enhanced hand hygiene and surface cleansing were also recommended. NPIs were gradually lifted from March 2021 onwards. Therefore, data were divided into pre-NPI (November 2017 to March 2020), NPI (April 2020 to March 2021) and post-NPI (April 2021 to December 2023) periods for analysis.

Our patient cohort was stratified into four age groups that aligned with the U.S. education/school system: preschool (0–5 years), elementary school (6–10 years), middle school (11–13 years), and high school (14–18 years). Co-infections with other respiratory pathogens were also evaluated. Descriptive statistics and Chi-square (χ^2^ independence) tests were used to calculate the test positivity rate (number of positive tests divided by all FARP performed) and the change in positivity rate among the pre-NPI, NPI, and post-NPI periods. Statistical analysis was performed using Microsoft Excel 365, with significance determined at a *P* value of <0.05. Descriptive statistics were used to calculate the test positivity rate (number of positive tests divided by all FARP performed) and interval change in the positivity rate (pre-NPI to NPI and NPI to post-NPI periods). Statistical differences were determined by chi-square (χ^2^ independence) analysis. All statistical analyses were performed with Microsoft Excel 365 (Microsoft Corp., Redmond, WA, USA); *P* values <0.05 were considered statistically significant.

## RESULTS

During the study, a total of 100,077 tests were performed on 56,834 unique patients 18 years old or younger. Among these patients, 46% were female. The tests were divided into three time periods: pre-NPI (8,972 tests), NPI (9,462 tests), and post-NPI (81,643). Total testing volume, over defined time periods, increased by 5.5% (pre-NPI to NPI), 763% (NPI to post-NPI), and 810% (pre-NPI to post-NPI). Table 1 provides an overview of the overall and age group-specific test positivity rates and the change in test positivity rates for the pre-NPI to NPI and NPI to post-NPI periods. The overall test positivity rate was 0.86%, 0.19%, and 0.03% for pre-NPI, NPI, and post-NPI periods, respectively. The overall test positivity rate decreased by 77% during the pre-NPI to NPI period; however, the positivity rate increased by 50% during the NPI to post-NPI period (*P* value <0.001).

**TABLE 1:** Summary of patient demographics, *M. pneumoniae* test positivity rate, and period-specific percent interval change

Demographic information		Pre-NPI period			NPI period			Post-NPI period			Period interval change	
		(11/2017–03/2020)			(04/2020–03/2021)			(04/2021–12/2023)			Pre-NPI→NPI	NPI→post-NPI
Age (year)	Unique Patients	Positive Tests	Total Tests	% Positive	Positive Tests	Total Tests	% Positive	Positive Tests	Total Tests	% Positive	% Change | *P* Value	
All	56,834	77	8,972	0.86%	18	9,462	0.19%	27	81,643	0.03%	−77 | <0.001	50 | <0.001
0–5	42,249	40	7,010	0.57%	11	6,395	0.17%	8	63,620	0.11%	−73 | <0.001	−27 | <0.001
6–10	8,765	24	881	2.72%	5	1,317	0.38%	11	10,332	0.11%	−79 | <0.001	120 | 0.012
11–13	3,263	7	441	1.59%	2	740	0.27%	4	3,470	0.12%	−71 | <0.011	100 | 0.310
14–18	4,047	6	640	0.94%	0	1,010	0.00%	4	4,221	0.09%	−100 | <0.003	>100 | 0.328

**Fig 1:**
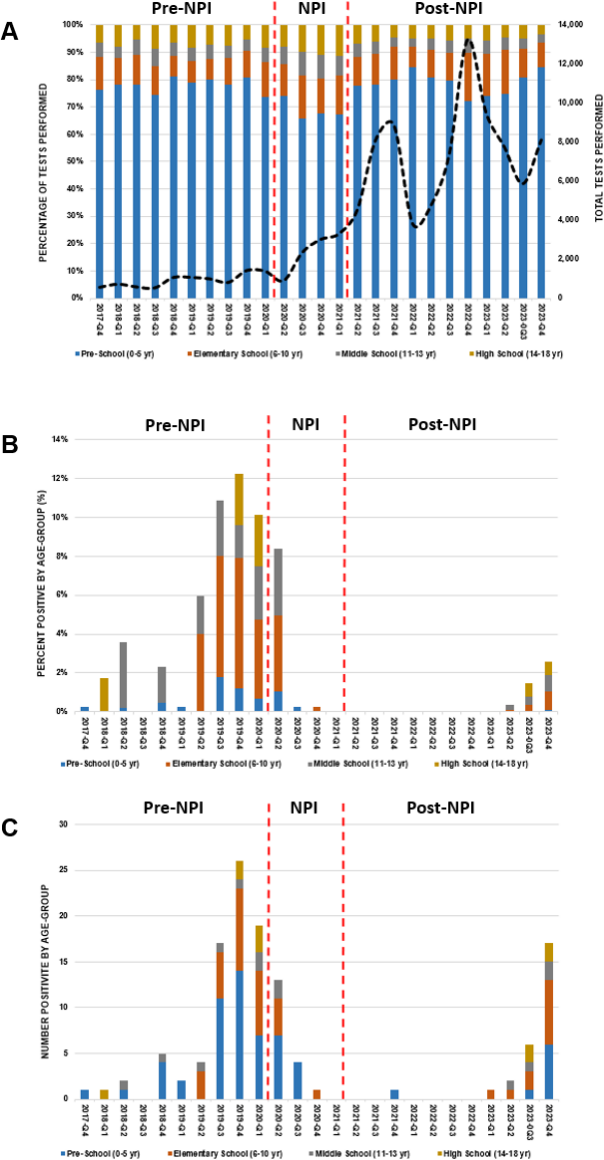
Summary of FilmArray Respiratory Panel testing. (Panel A) Total number of tests performed (black dashed line) and percentage of tests performed by age group. (Panel B) *M. pneumoniae* positivity rate by age group. (Panel C) Total number of *M. pneumoniae* positive tests by age group.

Considering the pre-NPI to NPI time periods, age group-specific *M. pneumoniae* test positivity rates changed as follows: preschool (73% decrease; *P* value <0.001); elementary school (79% decrease; *P* value <0.001); middle school (71% decrease; *P* value <0.001); and high school (100% decrease; *P* value <0.001). Considering the NPI to post-NPI time periods, age group-specific *M. pneumoniae* test positivity rates changed as follows: preschool (27% decrease; *P* value <0.001); elementary school (120% increase; *P* value <0.012); middle school (100% increase; *P* value <0.310); and high school (>100% increase; *P* value <0.328). However, the distribution of tests performed across different age groups remained relatively stable during the pre-NPI, NPI, and post-NPI periods as follows: preschool (78±2%, 69±4%, and 79±4%); elementary school (10±2%, 14±2%, and 12±3%); middle school (5±1%, 8±1%, and 4±1%); and high school (7±1%, 10±2%, and 5±1%) (Fig. 1A). The preschool group had the highest number of positive tests for *M. pneumoniae* (*n* = 59) and the highest age group-specific percent positivity rate (0.76%; 59/77,025 total tests). The elementary school group had the second highest number of positive tests for *M. pneumoniae* (*n* = 40) and the second highest age group-specific percent positivity rate (0.32%; 40/12,530 total tests). The middle school group had the third highest number of positive tests for *M. pneumoniae* (*n* = 13) and the third highest age group-specific percent positivity rate (0.28%; 13/4,651 total tests). The high school group had the lowest number of positive tests for *M. pneumoniae* (*n* = 10) and the lowest age group-specific percent positivity rate (0.17%; 10/5,871 total tests) (Table 1; Fig. 1B and C).

Table 2 highlights the differences in the number of co-infections (positive for *M. pneumoniae* and other upper respiratory tract viruses) during the pre-NPI (*n* = 36), NPI (*n* = 9), and post-NPI (*n* = 12) periods. Human rhinovirus/enterovirus was the most common co-infection among all age groups, primarily observed in the preschool and elementary school age groups. Respiratory syncytial virus, adenovirus, human metapneumovirus, parainfluenza virus, influenza virus, and non-SARS-CoV-2 coronaviruses were also identified as co-infections in specific age groups. A single co-infection with SARS-CoV-2 was observed in a middle school student. Co-infections with adenovirus, influenza, and non-SARS-CoV-2 coronaviruses were occasionally observed in high school students. Two or more co-infections were only observed in the preschool (*n* = 10) and elementary (*n* = 2) age groups (Table 2).

**TABLE 2:** Summary of co-infections (*M. pneumoniae* and respiratory viruses)

School group (Age)	Upper respiratory tract viral co-infections								Pre-NPI period	NPI period	Post-NPI period
	Mp	RV/EV	RSV	CoV	PIV	IFV	ADV	hMPV	(11/2017–03/2020)	(04/2020–03/2021)	(04/2021–12/2023)
**All**									**36**	**9**	**12**
Preschool (0–5 years)	X	X							10	2	5
	X	X	X						5	0	0
	X			X					2	0	0
	X		X		X				2	0	0
	X	X			X				1	3	0
	X		X				X		1	0	0
	X		X						1	0	1
	X					X		X	1	0	0
	X							X	1	0	0
	X						X		0	1	0
Elementary school (6–10 years)	X	X							4	2	0
	X						X		2	1	0
	X	X		X					2	0	0
	X		X						1	0	0
	X			X					0	0	1
	X							X	0	0	1
Middle school (11–13 years)	X	X							1	0	0
	X			X^*a*^					0	0	1
High school (14–18 years)	X	X					X		1	0	0
	X					X			1	0	0
	X	X							0	0	1
	X		X						0	0	1
	X			X					0	0	1

^*a*^ SARS-CoV-2; CoV, human coronaviruses (not SARS-CoV-2); ADV, adenovirus; IFV, influenza viruses; RV/EV, human rhinovirus/enterovirus; hMPV, human metapneumovirus; Mp; *M. pneumoniae*; PIV, parainfluenza viruses (1,2,3,4); RSV, respiratory syncytial virus.

**Fig 2:**
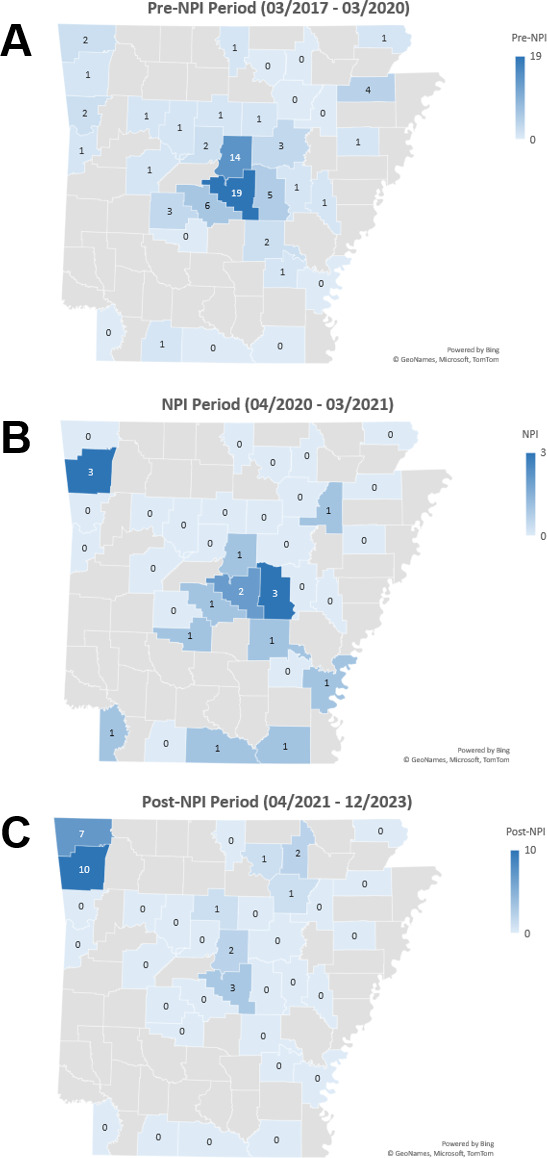
Number of positive tests for *M. pneumoniae* by county in Arkansas. The maps were created in Microsoft Excel (©GeoNames, Microsoft, TomTom).

Figure 2 illustrates the total number of positive tests for *M. pneumoniae* distributed among the 75 counties of Arkansas during the pre-NPI, NPI, and post-NPI periods. The data presented only represent residents of Arkansas who were tested using the FARP. Out of the total FARP tests performed (*n* = 100,077), 97.3% (97,454) were from Arkansas residents. The remaining 2.7% (2,623) were from individuals residing in neighboring states or traveling abroad when seeking healthcare. Only two non-resident Arkansans were positive for *M. pneumoniae* during the pre-NPI (*n* = 1) and NPI (*n* = 1) periods. Every Arkansas county was represented in the data set. The total number of tests performed by county ranged from 4 to 2,676 (mean = 13) during the pre-NPI, 2 to 3,123 (mean = 33) for the NPI, and 13 to 24,060 (mean = 89) during the post-NPI periods.

## DISCUSSION

In our study, which included just over 100,000 nucleic acid amplification tests (NAATs) from nearly 57,000 children representing every county in Arkansas, we observed an overall 77% reduction in the *M. pneumoniae* infection rate after NPI implementation. For the United States, our data can only be compared with two recent studies performed from April 2017 to March 2022 in Chicago, IL (15, 16). Using the same FARP test, these researchers demonstrated an 80% reduction in *M. pneumoniae* infection rates after implementation of NPIs (*n* = 20,751 NAATs). Other studies across the world have likewise shown decreases in *M. pneumoniae* infections. In a retrospective study from Children's Hospital Affiliated to Capital Institute of Pediatrics (Beijing, China), 569,887 pediatric patients with respiratory infections were studied from 01 June 2016 to 31 May 2021 (17). Using IgM-specific serology, the *M. pneumoniae* infection rate decreased from 17.59% in 2019 to 8.95% and 4.95% in 2020 and 2021, respectively. The significant decline in the *M. pneumoniae* infection rate was temporally associated with SARS-CoV-2 NPI implementation (17). Despite using different diagnostic testing modalities, reductions in *M. pneumoniae* infections were consistently observed; however, the magnitude of the reduction was most pronounced using NAAT vs. serology.

Our study also found that the reduction in *M. pneumoniae* infections was consistent across different age groups, including preschool, elementary school, middle school, and high school children. To our knowledge, these findings have not been previously reported in the United States. Similar findings have been documented in Israel, China, and Finland, where *M. pneumoniae* infection rates similarly decreased during the SARS-CoV-2 pandemic (14–21). A study from Henan Children's Hospital (Zhengzhou, China) evaluated 1,259,697 symptomatic children (≤18 years) from 2018 to 2021 (18). Using IgM-specific serology, the *M. pneumoniae* infection rate significantly decreased during the SARS-CoV-2 pandemic, and this was observed in all age groups (0–1 year, 1–3 years, 3–6 years, and 6–18 years) (18). Similarly, in Finland, the number of *M. pneumoniae* infections decreased by 72–89% during the SARS-CoV-2 pandemic when using NAAT; the decrease was consistently observed in children among the age groups 0–4 years, 5–9 years, and 10–14 years (19, 21).

Data regarding children co-infected with *M. pneumoniae* and respiratory viruses predate the SARS-CoV-2 pandemic and are mainly limited to children in the United States and China with CAP (22–28). These studies, conducted between 2010 and 2019 and involving approximately 3,000 children, showed that co-infections with respiratory viruses were common, ranging from 15% to 66% (22–28). As replicated in our study, co-infections with two or more respiratory viruses were primarily observed in young children. The most frequently observed viral co-infections included human rhinovirus/enterovirus, parainfluenza viruses, adenovirus, influenza viruses, respiratory syncytial virus, human coronaviruses (excluding SARS-CoV-2), human metapneumovirus, human bocaviruses, and parechoviruses. Our study observed co-infections with *M. pneumoniae* and respiratory viruses in both the pre-NPI, NPI, and post-NPI periods, with similar viral etiology and age group distribution. However, NPIs effectively reduced the transmission and acquisition of *M. pneumoniae* and most co-infecting respiratory viruses, except for human rhinovirus/enterovirus and adenovirus. It is worth noting that, during the SARS-CoV-2 pandemic, NPIs successfully reduced or temporarily eliminated most circulating respiratory viruses, except for human rhinovirus/enterovirus and adenovirus, which continued to spread at reduced levels (29). The reasons for this are not fully understood. Still, they may be related to factors such as asymptomatic carriage, unique transmission mechanisms, and the prolonged survival of these nonenveloped viruses on surfaces.

When considering all children spanning the NPI to post-NPI periods, there was a 50% increase (*P* < 0.001) in the *M. pneumoniae* infection rates (Table 1). Specifically, this increase was attributed to the last 6 months (July to December 2023) of our data (Fig. 1C). When stratified by age group, the elementary school (6–10 years), middle school (11–13 years), and high school (14–18 years) age groups each observed a 100% or greater increase in their respective *M. pneumoniae* infection rates. For the preschool (0–5 years) age group, we observed a 27% reduction in the *M. pneumoniae* infection rate; however, this reduction is markedly less than the 73% rate reduction observed for the same age group during the pre-NPI to NPI periods. These data suggest the emerging reestablishment of *M. pneumoniae* within our pediatric population.

Our study has some limitations. First, we did not correlate positive test results with clinical, radiographic, and other laboratory information, so we cannot determine if patients were infected or colonized by *M. pneumoniae* and/or other respiratory viruses. Second, our data represent an aggregated view and may not reflect specific individuals seeking medical care in different settings. Third, we could not verify the extent of NPI compliance among patients who underwent FARP testing during the SARS-CoV-2 pandemic, which may affect the accuracy of our data in assessing the actual effectiveness of NPI implementation in reducing *M. pneumoniae* and co-infecting respiratory viruses. Finally, we are not comparing the decreased *M. pneumoniae* infection rates across published studies that utilized different diagnostic testing methods (e.g., NAAT vs. serology). We are elaborating on the work of many researchers who have observed similar trends in the effectiveness of NPI implementation. We also highlight that the magnitude of measured changes depends on the testing modality utilized.

### Conclusions

Our data add to the growing body of evidence supporting the effectiveness of NPIs in reducing infections due to *M. pneumoniae* and co-infections with most respiratory viruses in pediatric patients in Arkansas. Specifically, co-infections with *M. pneumoniae* and human rhinovirus/enterovirus and/or adenovirus were still present, albeit at lower levels, during the SARS-CoV-2 pandemic. These findings suggest that NPIs alone do not effectively eliminate these specific viruses. Due to transmission mechanisms and the nonenveloped nature of both human rhinovirus/enterovirus and adenovirus, additional infection control measures (e.g., surface decontamination and improved hand hygiene) will likely be required to reduce their spread. With the relaxation of NPIs and the recent declaration of the end of the SARS-CoV-2 pandemic, respiratory viruses will continue to return to pre-pandemic levels and establish traditional circulatory patterns. It is well known that the replication time for viruses is exponentially faster than for bacteria, including *M. pneumoniae*. Therefore, based on our data, it is safe to assume that a resurgence of *M. pneumoniae* infections is currently in progress and will be important as it reestablishes itself within the human population.
